# Preparation and Representation of Recombinant Mn-Ferritin Flower-Like Spherical Aggregates from Marine Invertebrates

**DOI:** 10.1371/journal.pone.0119427

**Published:** 2015-04-16

**Authors:** Liping Chen, Jun Zhou, Yunyun Zhang, Shuangshuang Chu, Weina He, Ye Li, Xiurong Su

**Affiliations:** School of Marine Sciences, Ningbo University, Ningbo, Zhejiang Province, People’s Republic of China; CINVESTAV-IPN, MEXICO

## Abstract

Ferritin has important functions in the transition and storage of toxic metal ions, but its regulation and function in many invertebrate species are still largely unknown. In our previous work, the cDNA sequence of *Sinonovacula constricta*, *Apostichopus japonicas* and *Acaudina leucoprocta* were constructed and efficiently expressed in *E*. *Coli* BL21 under IPTG induction. In this follow-up study, the recombinant ferritins were exposed to heavy metal manganese. The manganese concentration levels in three recombinant ferritins were greater than horse spleen ferritin (HSF). Compared with HSF, the amount of manganese enrichment in the three recombinant ferritins was 1.75-fold, 3.25-fold and 2.42-fold increases in ScFER, AjFER, and AlFER, respectively. After phosphate stimulation, the concentration of manganese increased and was higher than the ordinary dialysis control groups. The ScFER was four times its baseline value. The AjFER and AlFER were 1.4- and 8-fold higher, respectively. The AlFER sample stimulated by phosphate was 22-fold that of HSF. The morphologies of the resulting Mn-Ferritin from different marine invertebrates were characterized with scanning electron microscopy. Surface morphologies were lamella flower-like and are consistent with changes in surface morphologies of the standard Mn-HSF. Invertebrate recombinant ferritin and HSF both can uptake manganese. We found that the structure of *A*. *leucoprocta*recombinant Mn-Ferritin aggregate changed over time. The surface formed lamella flower-like aggregate, but gradually merged to create a relatively uniform plate-like phase of aggregate spherically and fused without clear boundaries.

## Introduction

Ferritin is an iron-rich component first discovered in horse livers[1]. It is a highly conserved protein across animals, plants and microbes that stores and releases iron[2]. The first isolation of ferritin was facilitated by several distinct biochemical characteristics including its stability at high temperatures (80°C), relative insolubility in ammonium sulfate, and its crystallization with cadmium salts[3, 4]. Excess iron should be stored in ferritin to avoid iron poisoning. Ferritin releases iron to cells for bio-synthetic proteins or enzymes that utilize iron[5, 6]. Ferritin controls the amount of iron in the organism and has detoxification properties[7].

Ferritin exists in a roughly spherical configuration via the assembly of 24 subunits from two types of polypeptide chains. The structure controls its functions[8]. The H chains catalyze the first step in iron storage and the oxidation of iron(II). The L chains promote the nucleation of the mineral ferrihydrite and enable storage of iron(III) inside the protein shell[9, 10]. Ferritin can use a three-phase tunnel (X, Y, Z) on the protein shell as a internal exchange channel to participate in the release and storage of iron[11]. Indeed, natural ferritin can store toxic metal ions including Zn^2+^, Pb^2+^, and Ni^2+^[12]. Studies suggested that the prokaryotic expression of recombinant ferritin from *Dendrorhynchus zhejiangensis* had the ability to enrich divalent metal ions including Cd^2+^, Pb^2+^, and Fe^2+^[13]. The metal sequestering capacity of ferritin had even been used for the size-selective synthesis of many metal nanoparticles[14, 15]. Moreover, the conductivity of the ferritin were directly influenced by the metal cores as Mn(III)-ferritin, Co(III)-ferritin, and Cu(II)-ferritin, which could be highly relevant in realizing metalloprotein-based bioelectronic devices[16]. According to research Mn(III) oxyhydroxide (MnOOH) cores within the nanoscale cavity of the iron storage protein horse spleen ferritin could be successfully established[17]. Study also found that Fe^3+^ chelated monosize nanospheres used in ferritin adsorption could greatly increase the ferritin adsorption capacity, and the adsorption behavior of ferritin could be modeled using both Langmuir and Freundlich isotherms[18]. Furthermore, the relative changes in the different metals were reflected in the contributions of the metal core reconstituted ferritins[19]. Clear distinctions could be made among the voltage responses of the metallic gold substrate surface through the metal-containing ferritin form and the metal-free ferritin form[20].

In contrast to the plethora of studies on vertebrate ferritins, there were relatively few about invertebrates. Ferritins from bumblebee[21], amphioxus[22], freshwater giant prawn[23], freshwater crayfish[24], snail[25], and pearl oyster[4] showed largely different sequences with identities as low as 15%. However, the three-dimensional structures were remarkably well conserved[26]. They are all quite similar. These features are helpful to the further development and utilization of ferritin. To date, our lab has analyzed the gene sequence from *Sinono vaculaconstricta*, *Apostichopus japonicas*, and *Acaudina leucoprocta* ferritin. These three invertebrates are the most important aquaculture products on the Chinese coast, but the waters are polluted with heavy metals and organic compounds. These species have some protection from environmental stressors, and ferritin is an important resistance gene. Ferritin may chelate and sequester these toxins to prevent some negative effects. It is also the key and foundation of improved variety breeding. However, enrichment of manganese by these three invertebrate recombinant ferritins has not yet been studied.

Manganese is a trace element ubiquitously distributed throughout surface soils, aquatic sediments and ground waters. The amount of the manganese in environment gradually increased with metal mining and other industrial activities[27, 28]. Human exposure to manganese has increased, increasing the potential risk of manganese poisoning. The toxic effects of manganese are nervous system abnormalities. Chronic manganese poisoning results in pyramidal nerve dysfunction similar to the symptoms of Parkinson’s disease, respiratory problems, bronchitis, and pneumonia [29–31].

Beyond the toxic effects of manganese, humoral and cellular immunity following manganese treatment in mice had been described previously[32]. However the enrichment of these interactions with ferritin has not yet been attempted. Characterization of the manganese core of reconstituted ferritin by X-ray absorption spectroscopy had been shown, but the specific enrichment of manganese and surface properties of remained unexplored[33]. Barindra *et al*. showed that the nanocomposite contains ferritin and manganese showed high relaxivity that also indicated its utilityas an ultrasensitive materials or reagents[34].

Using ferritin to remove manganese from water can reduce the cost of secondary pollution. The study has great potential applications for the purification of manganese. The hollow spherical shells of ferritin sequestered and maintained iron in a nontoxic and bio-available form[35]. The ferritin enriched manganese and might remove manganese pollution from the environment.

In this study, recombinant *S*. *constricta*, *A*. *japonicas*, and *A*. *leucoprocta* ferritin were expressed in cDNA sequence previously cloned in our laboratory. They were purified and used for enrichment of manganese.

## Materials and Methods

### The preparation of recombinant ferritins

In our previous work, we cloned inducible ferritin cDNAs from *S*. *constricta*, *A*. *japonicus*, and *A*. *leucoprocta*[36–38]. The PCR products of *S*. *constricta*, *A*. *japonicus*, and *A*. *leucoprocta* ferritins as well as the pET-28a plasmid were digested by *Bam* H I and *Hind* III. These were then ligated with the T4 DNA ligase. The recombinant plasmid pET-FER was then transformed into *E*. *coli* BL21 strain and grown on agar plates with ampicillin. Plasmid DNA extracted from antibiotic resistant clones was identified by enzyme digestion. A positive clone was cultured overnight at 37°C, and then diluted 1:100 into LB broth. The bacterial growth was monitored by measuring the cell densities at OD_600_. When the OD_600_ was between 0.6 and 0.8, IPTG was added at 1 mmol L^-1^. The culture was continued for another 1–5 h. The ferritin was purified using a Ni-NTA affinity column (GE healthcare) according to the manufacturer’s instruction. The purified proteins were refolded against stepwise decrease of urea with the order of 6 M, 4 M and 2 M concentration in GSH/GSSG buffer (50 mM Tris-HCl, 1 mM EDTA, 50 mM NaCl, 10% glycerol, 1% glycine, 2 mM reduced glutathione, 0.2 mM oxide glutathione, pH 8.0) overnight at 4^°^C. This allowed analysis of protein refolding and gave recombinant ferritin termed ScFER, AjFER, AlFER from *S*. *constricta*, *A*. *japonicus*, and *A*. *Leucoprocta*, respectively. The concentration of the purified protein was quantified by the BCA Method (Nanjing Jiancheng Bioengineering Institute). The expression and purified products were separated by SDS-PAGE and stained with Coomassie brilliant G250.

### Heavy metal manganese treatment

#### Manganese treatment

To determine heavy metal enrichment capacity 2 mL of ScFER, AjFER, AlFER, and horse spleen ferritin (HSF) (Sigma) were dialyzed with 100 mL of 2 mM MnCl_2_ (pH 8.0) at 4°C for 12 h. The sample was then dialyzed in 2 mM Tris-HCl to remove free Mn^2+^. Dialysis used magnetic stirring, and the solution was replaced every 4 h.

#### Phosphate stimulation

After exposing the ScFER, AjFER, AlFER, and HSF to 2 mM MnCl_2_ for 12 hours, the dialysis solution was replaced with 2 mM phosphate buffer saline (PBS) stimulating environmental dialysis. It was finally dialyzed with 2 mM Tris-HCl to remove free Mn^2+^.

### Detecting manganese by ICP-MS

The 35 samples of ferritin enriched with manganese were collected from dialysis. There were 5 samples from each of the 7 ferritin species: ScFER, ScFER stimulated by phosphate, AjFER, AjFER stimulated by phosphate, AlFER, AlFER stimulated by phosphate, HSF.

For microwave digestion, 1 mL of each ferritin sample was analytically transferred into a PTFE digestion vessel. Then, 7 mL of concentrated HNO_3_ (70%) and 1.0 mL H_2_O_2_ was added and digested under microwave (MARS, CEM, NC, USA). The combustion procedure was as follows: (1) 1000 W at 80°C for 5 min, (2) 1000 W at 50°C for 5 min, (3) 1000 W at 190°C for 20 min, and (4) 0 W for 30 min for cooling. After cooling, the samples were transferred to 50 mL self-standing polypropylene volumetric tubes with plug seal caps. The contents were diluted to 25.0 g with ultrapure deionised water and studied with inductively coupled plasma-mass spectrometry (NexION 300X, PerkinElmer, New York, USA). Sample blanks were prepared using the full analytical procedure except addition of ferritin samples[39]. The calibration standard solutions were 10 mg/L multi-element standard solution (CLMS-2AN, Spex, CA, USA), Standard Solution of Stannum element (100mg/L, GBW(E)080546, Beijing Century Aoke Biotechnology, China).

### Observation of ferritin aggregates by SEM-EDS

Surface morphologies were observed using scanning electron microscopy (SEM) (S-3400N, Hitachi, Japan) and Energy Dispersive Spectrometry (EDS). A 0.5 mL aliquot of each ferritin (ScFER, AjFER, AlFER, and HSF) was evenly distributed on the surface of a pretreated mica sheet and dried for 4 days at room temperature at 5% humidity. In addition, 15 groups each with 3 replicates were evenly distributed on the surface of pretreated mica sheet and dried. These samples were 0.5 mL of AlFER protein (a kind of random selection) dripped on the surface. We selected one group and observed the surface morphology daily.

## Results

### Expression and purification of recombinant ScFER, AjFER, AlFER proteins

**Fig 1:**
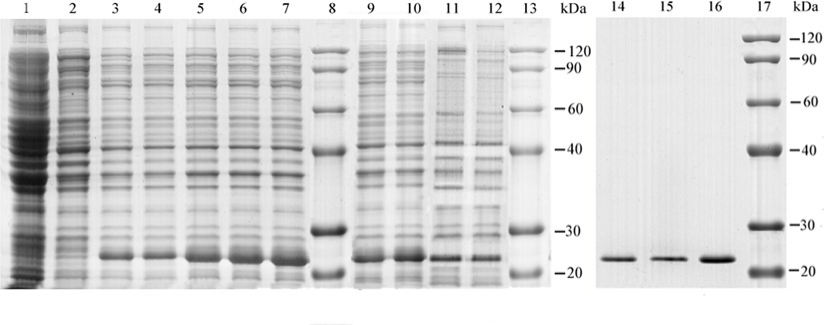
Expression and purification of pET-FER recombinant ScFER, AjFER, AlFER protein in *E*. *coli* strain BL21. Lanes 8, 13, and 17: middle molecular marker; lanes 1 and 2: negative control; lanes 3–7: ScFER induce expression at 1, 2, 3, 4, and 5 h; lanes 9 and 10: AjFER expression at 4 and 5 h; lanes 11 and 12: AlFER induce expression at 4 and 5h; lanes 14–16: AlFER, AjFER, and ScFER purified expression products.

The expression products were analyzed with SDS-PAGE to characterize the recombinant ScFER, AjFER, and AlFER. After IPTG induction, clear protein bands with molecular weights between 20 and 30 kDa were detected in the positive transformants and were purified to homogeneity (Fig 1). The molecular mass of the purified product was in good agreement with the predicted molecular weight of ScFER, AjFER, and AlFER. These three protein bands had molecular weights between 20.1 and 29 kDa and were detected in the positive transformants in our previous work[36–38]. Here, recombinant ferritin from *S*. *constricta*, *S*. *japonicus*, and *A*. *Leucoprocta* were expressed and purified as experimental materials, and the correctness was verified by western blot in the past[36–38]. The intensity of the recombinant protein bands increased with time, and peak expression of recombinant ferritin occurred 5 h after IPTG was introduced.

### Manganese Energy Detection

**Fig 2:**
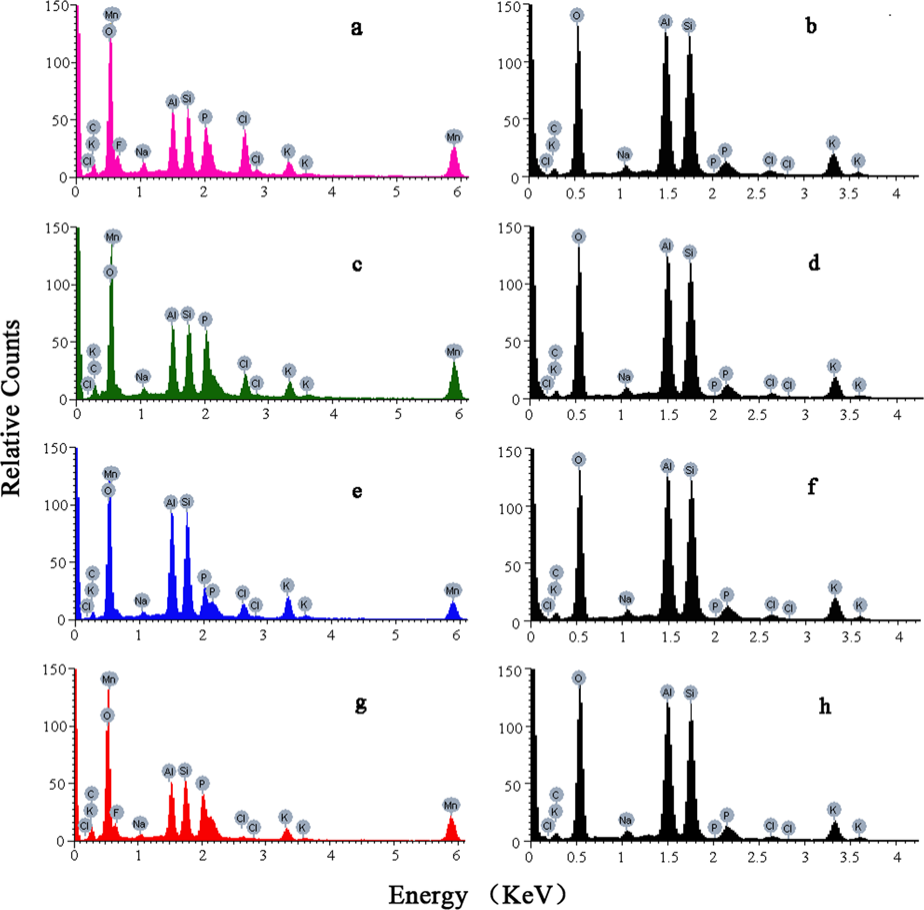
EDS results of different ferritin treatment groups. Peaks of manganese for each ferritin can be clearly detected in the same position (0.56 and 5.91 keV) in the manganese treatment groups (a, c, e, g). No manganese peak was detected in the negative controls (b, d, f, h). a: ScFER with manganese treatment; b: the negative control of ScFER without manganese treatment; c: AjFER with manganese treatment; d: the negative control of AjFER without manganese treatment; e: AlFER with manganese treatment; f: the negative control of AlFER without manganese treatment; g: HSF with manganese treatment; h: the negative control of HSF without manganese treatment.

EDS confirmed that ferritin trapped manganese. Manganese was seen in the EDS data and qualitatively confirmed that ferritin bound manganese. Controls included four kinds of ferritins without manganese treatment. Elemental analysis of eight kinds samples were shown in Fig 2. All the manganese-treated groups showed manganese, but the control groups did not. The Si, Al, F and Cl peaks were from the mica sheet. The C, O, and P were from ferritin and are a vital component of the active center group of that chelates iron and heavy metals. The C = O, P = O, P-OH, P-O-P, and P-O-C sites were manganese enrichment groups. Elements detected in the control group of different ferritins were basically the same intensity. This confirmed that the composition of the different recombinant ferritin was relatively stable and similar to HSF. Different recombinant ferritin samples had different Mn values(Fig 2), because of different rates of metal enrichment.

Manganese had fixed electron transition levels. There were four shells including K, L, M, and N outside the Mn atomic nucleus. After irradiation, different shells outside the core of the electronic transition released different energies. Thus, there were different energy X-ray spectra with different peaks. The recombinant ferritins and HSF contained manganese at 0.56 and 5.91 keV. The EDS showed that Mn had entered ferritin through dialysis.

### Phosphoric acid effect of capacities to uptake manganese of recombinant ferritins

**Fig 3:**
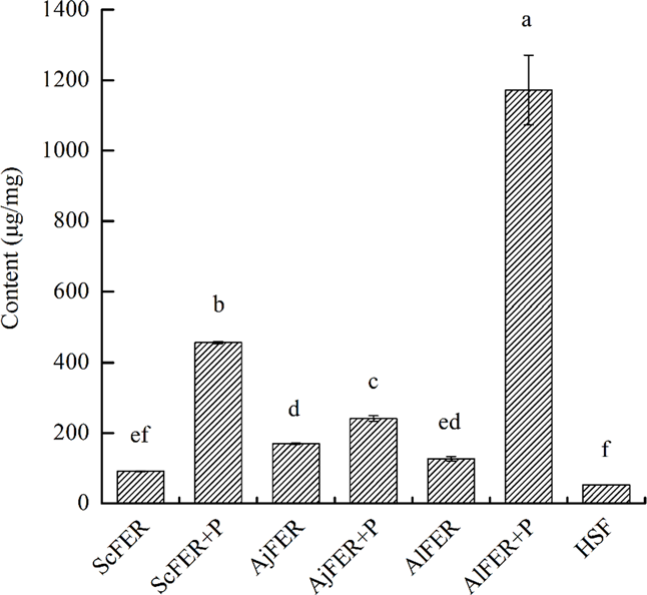
The content of manganese in different ferritin groups. AlFER+P: AlFER with phosphate-stimulated, AjFER+P: AjFER with phosphate-stimulated, ScFER+P: ScFER with phosphate-stimulated. Each symbol and vertical bar represented the mean ±S.D (n = 5). Significant differences between challenged groups were indicated by letters. Different letters represent significant difference, the same letters represent no significant difference (P<0.01).

EDS is qualitative. Thus, we used ICP-MS to further confirm that manganese entered ferritin mineral core. Our goal was to show sensitivity, selectivity and multielement analysis capability. The data in Fig 3 showed that the concentration of Mn in ScFER, AjFER, and AlFER were higher than in standard HSF (P<0.05). Versus HSF, the enrichment levels of manganese in the three recombinant ferritins were 1.75-fold, 3.25-fold and 2.42-fold higher in ScFER, AjFER, and AlFER, respectively.

Meanwhile, the phosphate-stimulated groups had higher manganese concentration than ordinary dialysis control groups (P<0.05). The ScFER was four times its baseline value. The AjFER and AlFER were 1.4- and 8-fold higher, respectively. The AlFER sample was 22-fold that of HSF.

### Morphology of recombinant Mn-Ferritin aggregates

**Fig 4:**
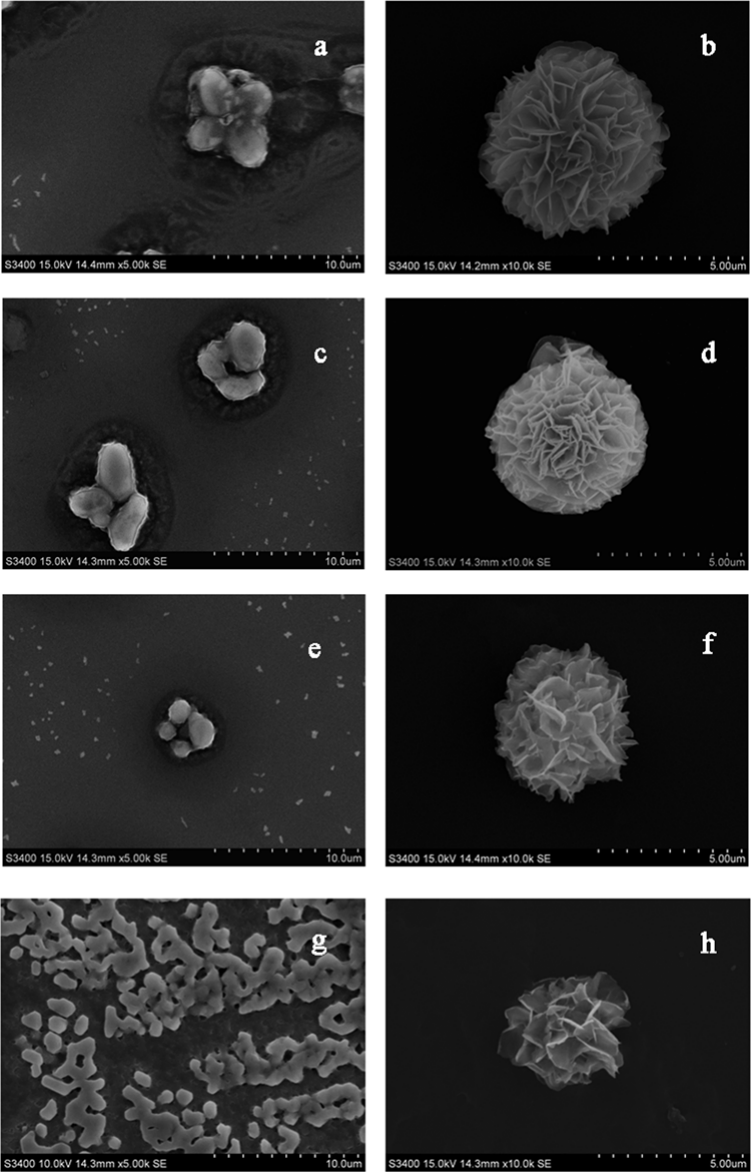
SEM of different treatment groups of ferritin. a: ScFER aggregates morphology; b: Mn-ScFER aggregates; c: AjFER aggregates morphology; d: Mn-AjFER aggregates; e: AlFER aggregates morphology; f: Mn-AlFER aggregates; g: HSF aggregates morphology; and h: Mn-HSF aggregates.

The EDS and ICP-MS suggested that ferritin bound manganese. The ferritin protein cage can remain assembled and has an altered metal mineral phase after chelation[35]. Scanning electron microscopy (SEM) also studied the Mn-Ferritin aggregates surfaces. The results in Fig 4a, 4c and 4e showed that the aggregates of ScFER, AjFER, and AlFER without metal dialysis treatment had relatively similar cage-like surface morphologies with separated spherical shapes.

The aggregate created a large cavity that may accommodate the manganese core. In Fig 4g the HSF aggregates were similar and range from 1 to 2 μm. The HSF aggregates closed to each other and formed lattices. In contrast to the negative control, the aggregates of ScFER, AjFER, AlFER, and HSF with manganese treatment formed Mn-Ferritin aggregates of relatively uniform lamella flower-like spherical shape (Fig 4b, 4d, 4f, and 4h). These flower-like spherical structures were ~ 5 μm. This confirmed that manganese interacted with the invertebrate ferritins. The Mn created Mn-Ferritin aggregates and changed the surface morphology. The similar surface characteristics of Mn-Ferritin aggregates indicated that recombinant ferritins as ScFER, AjFER, and AlFER shared common physical and chemical properties to HSF from higher vertebrate animal. Manganese had the same chemical changes in these ferritin samples.

**Fig 5:**
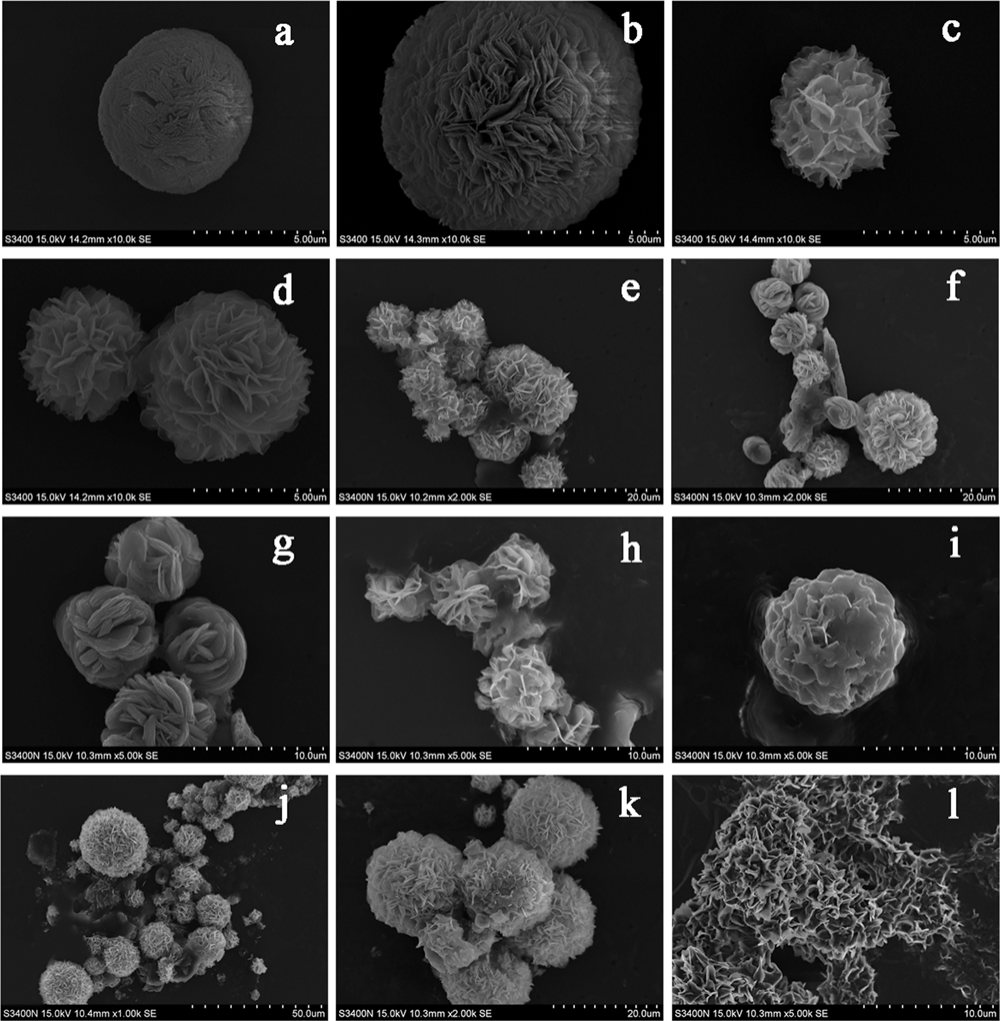
The surface characteristics of Mn-AlFER aggregates. a: surface observed after 1 day; 5: surface observed after 2 days; c and d: surface observed after 5 days; e: surface observed after 6 days; f and g: surface observed after 7 days; h and i: surface observed after 9 days; j and k: surface observed after 12 days; l: surface observed after 15 days.

The surface of AlFER aggregates was observed by SEM to find a mechanism of Mn-Ferritin aggregates evolution. The samples were stored at room temperature. The surface morphology of the Mn-Ferritin aggregates changed constantly and showed a growing, blooming state shaped like a spherical flower. After one day (Fig 5a), the surface of the Mn-Ferritin aggregate had a regular morphology. It was a single sphere with a densely gathered sheet. After two days (Fig 5b), the Mn-Ferritin aggregate surface layer exhibited a dense needle-like sheet bloom state, and the gap between the sheets increase. After five days (Fig 5c and 5d), the surface aggregate diverging lamellar petals, and the gap between the sheets increases. Aggregates appeared as single spheres or closely associated pairs. The configurations of surface were basically the same. Samples at day six were shown in Fig 5e. Multiple spheres joined to create larger aggregates. The lamellar petals were shaped and spread evenly like a lamella flower. Multiple round spheres joined, but the lamellar petals on the surfaces became thicker and wider after seven days (Fig 5f). Some lamellar petals on the surface of the sphere became more structured and changed morphology (Fig 5g). Part of the aggregates surface layers gradually merged and became smooth after nine days (Fig 5h). Fig 5i showed the layers on the surface of some scattered single sphere aggregates fused. The sphere surface was concave and convex shaped with irregular pores instead of the original sharp lamellas. After twelve days (Fig 5j), the Mn-AlFER aggregates became denser and more spherical. Spheres in bigger aggregates with different sizes had different surface morphologies. Some had acicular layers, some had fusion layers, and some were concave or convex shapes. Larger globular aggregates could also be observed (Fig 5k). The boundary was not very clear between the different spheres; it increasingly merged. After 15 days, the Mn-Ferritin aggregates with a dense surface layer became more intense and the boundary nearly disappeared (Fig 5l). Surface layer thickness was not consistent. The directions of the lamella curls were irregular with some pores.

**Fig 6:**
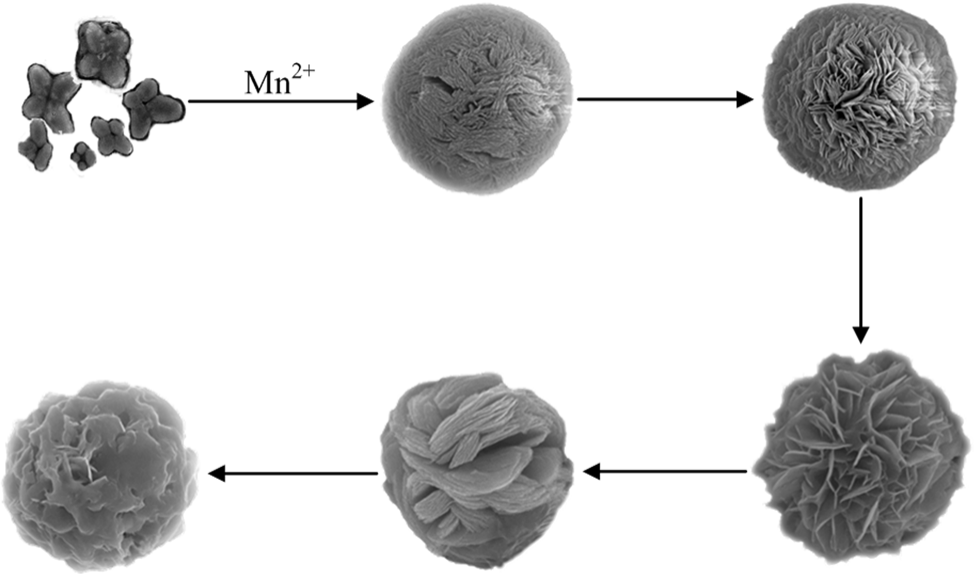
The evolution of Mn-AlFER aggregates.

Mn-Ferritin aggregate with a spherical, lamella flower-like morphology formed after addition of Mn. It grew and changed at room temperature after manganese treatment. It was clear that the morphology grew for Mn-Ferritin aggregates (Fig 6) even without considering late stage aggregate adhesion. The surface morphology of ferritin that had not enriched manganese was not particularly round. After enrichment of manganese, ferritins showed globular aggregates with constantly changing surface morphology. The macro-structure looked like a spherical flower. After enrichment of manganese, ferritins aggregated with 2 spheres or 3 spheres together unceasingly. Ferritin aggregates gradually became larger pieces with gathered sphere boundaries and little space between points.

## Discussion

Three kinds of recombinant ferritins were cloned and prokaryotic expressed according to the gene sequence from *S*. *constricta*, *S*. *japonicus*, and *A*. *leucoprocta* [36–38]. These three benthic animals are widely distribution in coastal areas of China. The ferritins which were critical to participate in the physiological function had high homology in the bodies[1, 2]. Their interiors had no iron core frameworks supporting[36, 37]. Recombinant ferritins which were more flexible with higher plasticity mean new empty cavities, could maximize the adsorption of manganese[40]. The HSF came from biological extraction must have bound irons in organisms before it was extracted, which had no sufficient space to bind more extra manganese. Its elasticity and plasticity may be worse than the other three recombinant ferritins. Less stretching space decreased manganese uptake.

The mechanism of ferritin uptake and its binding to manganese was very complicated. Heavy metal binding sites include internal and external surfaces of the protein shell as well as three-phase, two-phase, iron core surface and the deep tunnel[41, 42]. Phosphate was involved in the formation of the iron core, and it could be released from ferritin when the iron core was chemically reduced[43]. Phosphate acted as a chelating agent in ion exchange from ferritin[44]. Negatively charged phosphate groups could increase the electrostatic repulsion between ferritin molecules and make it easier to disperse in solution—this decreases the surface tension[45]. Elemental analysis also revealed that approximately 80% of the Fe(II) was removed from ferritin when the ferritin was reduced in the presence of phosphate[46]. Also, phosphoric acid could strengthen the electrochemical signals and enrich metal ions in the iron core[47].

The metal phosphate seed itself was an autocatalyst, and it would form quickly in the ferritin cavity[47]. The manganese seeds first formed in the inner surface of the cavity as a function of manganese concentration. Phosphate ions inside the protein cavity combined with manganese to form metal phosphates. The metal phosphates could keep binding the metal cations and removing phosphate radicals[47, 48]. The manganese ion and phosphate outside the cavity entered the nucleus through the tunnel until the core was filled. Phosphate enhanced the ferritin enrichment of manganese.

Manganese has a strong oxidation/reduction function. A deep-sea manganese-oxidizing bacterium (strain Mn32) oxidizes soluble Mn(II) to insoluble biogenic Mn oxides birnessite and manganite. Biogenic Mn oxides were further adsorbed by Mn(II) from the culture[49]. The study of deep-sea manganese oxide bacteria Mn32 found that the surface morphology of biogenic Mn oxides was layered or flaked[50, 51]. Mn-Ferritin aggregates had a relatively uniform plate-like spherical shape similar to Mn32.

Manganese oxide is the primary unit of octahedral molecular sieves. Its secondary structure consists of total points, edges or coplanar connections of manganese oxide into octahedral chains or belts[52]. This structure easily combines with other ions and also changes the manganese oxide material structure. It is easily formed from the structure of porous manganese oxide[53]. Manganese oxide with octahedral surface layers regulates the reaction performance via manganese valence electrons or electron replacement[54]. The adaptability of manganese oxide made it important in research of adsorbents, batteries, catalysts, carriers, detergents, sensors, etc.[55, 56]. The EDS analysis showed Mn on the surfaces of the Mn-Ferritin aggregates. There might be catalytic factors similar to multicopper oxidases that can promote the reaction. Studies showed that multicopper oxidases catalyze the microbial oxidation of manganese[50, 57]. Each enzyme had a high specificity towards iron with the resulting ferroxidase activity associated with a ferro-protein[58].

Ferritin derived from invertebrates, plants, microorganisms contain only one subunit that was very similar to the mammalian H subunit[8]. The physiological function of invertebrate ferritin was the same as two subunits from mammal. The ferritin enzyme activity associated with iron oxidation also assisted invertebrate ferritin[5].

Fe^2+^ ions were oxidized to Fe^3+^ when they bound to the ferroxidase center in the presence of oxygen[59]. The Fe^3+^ ions migrated to the interior of the ferritin from the ferroxidase center after oxidation. After the formation of a metal core, the iron ions became oxidized directly on the mineral core surface with migration through the 3-fold channels[60]. Iron oxidation on the core surface was faster than at the ferroxidase center. The ferroxidase center still functioned after the core was established, and its contribution to ion oxidation was less significant than oxidation on the mineral core surface[61].

There were electronic and materials tunnels in the ferritin shell[62]. Electronic tunnels consisted of some amino acid residues and were responsible for electronic transfer, storage and release of irons[63, 64]. Analysis of anion loading into ferritin after the iron core was established that allowed the anions to deposit on the mineral core. This suggested that the phosphate groups were important for ion release[65, 66]. This co-deposition lead to simultaneous deposition of the cation and anion and formed a circle that could contribute to the enrichment of metal ions. Treffry pointed out that iron storage by ferritin may utilize double iron cluster structures and catalytic conversion of Fe^2+^ into Fe^3+^[40]. Similar adsorption capacities existed for biogenic Mn oxides produced by Mn32. This ferritin may share some similar physical and chemical properties in the enrichment of manganese.

The lamella flower-like Mn-ferritin aggregates are another future direction. It is still unknown what percentage of the ferritin is actually holding manganese and how that changes as a function of time, temperature, and pH. The function of the Mn-Ferritin particles is also a focus in the future researches. Finally, potential interactions and synergies between manganese and other metals deserve future work.
